# Quantitative Changes in the Transcription of Phytohormone-Related Genes: Some Transcription Factors Are Major Causes of the Wheat Mutant *dmc* Not Tillering

**DOI:** 10.3390/ijms19051324

**Published:** 2018-04-29

**Authors:** Ruishi He, Yongjing Ni, Junchang Li, Zhixin Jiao, Xinxin Zhu, Yumei Jiang, Qiaoyun Li, Jishan Niu

**Affiliations:** 1 National Centre of Engineering and Technological Research for Wheat/Key Laboratory of Physiological Ecology and Genetic Improvement of Food Crops in Henan Province, Henan Agricultural University, Zhengzhou 450046, Henan, China; guomai301@163.com (R.H.); chang_top@163.com (J.L.); zxjiao2018@163.com (Z.J.); xzx1202@126.com (X.Z.); xinnercw@126.com (Y.J.); lqylhy@163.com (Q.L.); 2 Shangqiu Academy of Agricultural and Forestry Sciences, Shangqiu 476000, Henan, China; nyj317@163.com

**Keywords:** wheat (*Triticum aestivum* L.), *dmc* mutant, transcriptome, tillering, differentially expressed genes

## Abstract

Tiller number is an important agronomic trait for grain yield of wheat (*Triticum aestivum* L.). A dwarf-monoculm wheat mutant (*dmc*) was obtained from cultivar Guomai 301 (wild type, WT). Here, we explored the molecular basis for the restrained tiller development of the mutant *dmc*. Two bulked samples of the mutant *dmc* (T1, T2 and T3) and WT (T4, T5 and T6) with three biological replicates were comparatively analyzed at the transcriptional level by bulked RNA sequencing (RNA-Seq). In total, 68.8 Gb data and 463 million reads were generated, 80% of which were mapped to the wheat reference genome of Chinese Spring. A total of 4904 differentially expressed genes (DEGs) were identified between the mutant *dmc* and WT. DEGs and their related major biological functions were characterized based on GO (Gene Ontology) and KEGG (Kyoto Encyclopedia of Genes and Genomes) categories. These results were confirmed by quantitatively analyzing the expression profiles of twelve selected DEGs via real-time qRT-PCR. The down-regulated gene expressions related to phytohormone syntheses of auxin, zeatin, cytokinin and some transcription factor (TF) families of TALE, and WOX might be the major causes of the mutant *dmc,* not tillering. Our work provides a foundation for subsequent tiller development research in the future.

## 1. Introduction

Wheat (*Triticum aestivum* L.) is one of the most important food crops in the world. Since tiller number is an important agronomic trait for grain yield [1,2], it has always been one of the key traits to select in breeding programs. Generally, low and high tillering wheat mutants do not have very high grain yield. Moreover, tiller number as well as grain number and weight affect yield.

Mutants with various tillering abilities are ideal materials for the study of tiller developmental molecular mechanisms. Four tiller inhibition lines or mutants have been reported in wheat. Among the tiller inhibition genes, *tin1*, *tin3* and *ftin* are recessive, and they have been mapped on chromosome (Chr.) 1AS [3], 3A [1] and 1AS [4], respectively; *tin2* is a dominant gene mapped on Chr. 2A [5]. One wheat high tillering mutant harbors a major quantitative trait locus (QTL; QHt.nau-2D) on Chr. 2DS [6]. In barley (*Hordeum vulgare*), the tiller inhibition genes *lnt1*, *als1*, *cul4*, *int-b* and *uzu* have been reported, and they have been mapped on Chr. 3HL [7], 3HL [8], 3HL [9], 5HL [9] and 3 HL [9], respectively. Many tiller related mutants have also been reported in rice (*Oryza sativa*) [10,11,12].

Tillering is a very complex trait; in addition to genetic factors, it can also be significantly affected by soil fertility and other environmental factors. Many studies showed that tillering is controlled by QTLs in wheat [13,14,15,16], rice [17,18,19], barley [20,21] and rye (*Secale cereale*) [22]. However, it is clear that some tiller traits are controlled by qualitative genes as described above. In Arabidopsis, the *MORE AXILLARY GROWTH 1* (*MAX1*) genes encoding cytochrome P450 and *MAX2* control shoot lateral branching [23,24]. The *MAX4* genes encoding CAROTENOID CLEAVAGE DIOXYGENASE 8 (CCD8) and *MAX3* genes encoding CCD7 regulate shoot branching [25,26]; *MAX1* acts downstream of MAX3 and MAX4. The *MAX1* genes are related to the strigolactone (SL) signaling pathway [24]. In comparison to the model species Arabidopsis, only a few genes affecting tiller initiation and outgrowth have been cloned and described in crops. For example, the rice gene *MONOCULM 1* (*MOC1*) encodes a GRAS (GIBBERELLIC-ACID INSENSITIVE (GAI), REPRESSOR of GAI (RGA) and SCARECROW (SCR)) domain-containing protein that affects the initiation and outgrowth of axillary meristems [27,28]. Mutant lines that do not have a functional *moc1* gene exhibit a severe reduction in tiller number. The rice gene *Tillering and Dwarf 1* (*TAD1*), which encodes a multi-subunit E3 ligase, regulates rice tillering by degenerating *MOC1* [29]. The rice *TEOSINTE BRANCHED 1* (*OsTB1*) gene encodes a TCP domain protein and negatively regulates lateral branching [30]. In barley, the *INTERMEDIUM-C* (*INT-C*) gene is an ortholog of maize and rice *TB1* genes which has an effect on seedling tiller number [31]. The barley *uzu* gene encodes a putative brassinosteroid (BR) receptor HvBRI1 and regulates tiller number [32]. In bread wheat, overexpression of tae-miR156 significantly affects tillering, probably by regulating a group of *SQUAMOSA PROMOTER BINDING PROTEIN-LIKE* (*SPL*) genes; furthermore, miR156-TaSPLs and strigolactone signaling pathways might have a potential association with tillering [33]. Overexpression of the maize *tb1* gene in wheat results in reduced tillering [34]. The molecular mechanism of wheat tillering remains largely unknown.

At present, the RNA-Sequencing technique has been widely used to identify differentially expressed genes (DEGs) among various biological samples, so as to explore the possible mechanisms leading to various morphologies [35,36]. Transcriptome analyses regarding tiller developments in switchgrass (*Panicum virgatum* L.) and sorghum (*Sorghum bicolor* L.) have been reported [37,38,39]. In this study, a wheat mutant that does not develop tillers was obtained from the ethyl methanesulfonate (EMS)-treated wheat cultivar Guomai 301 (WT), which was designated as *dmc* (dwarf-monoculm). We characterized the mutant *dmc* and WT at the transcriptome level by RNA-sequencing technology.

## 2. Results

### 2.1. Morphology of the dmc Mutant

**Figure 1:**
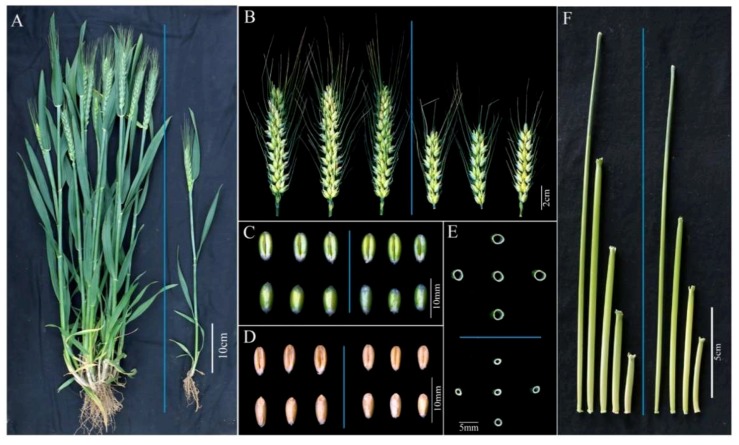
Comparison of agronomic traits between the WT and mutant *dmc*. (**A**) The plant phenotype of the WT (left) and mutant *dmc* (right); (**B**) the spikes of the WT (left) and mutant *dmc* (right); (**C**) the seeds of the WT (left) and mutant *dmc* (right) at the filling stage; (**D**) the seeds of the WT (left) and mutant *dmc* (right) at the mature stage; (**E**) the transverse sections of the top first internodes of the WT (up) and mutant *dmc* (down); and (**F**) the internodes of the WT (left) and mutant *dmc* individuals (right).

**Figure 2:**
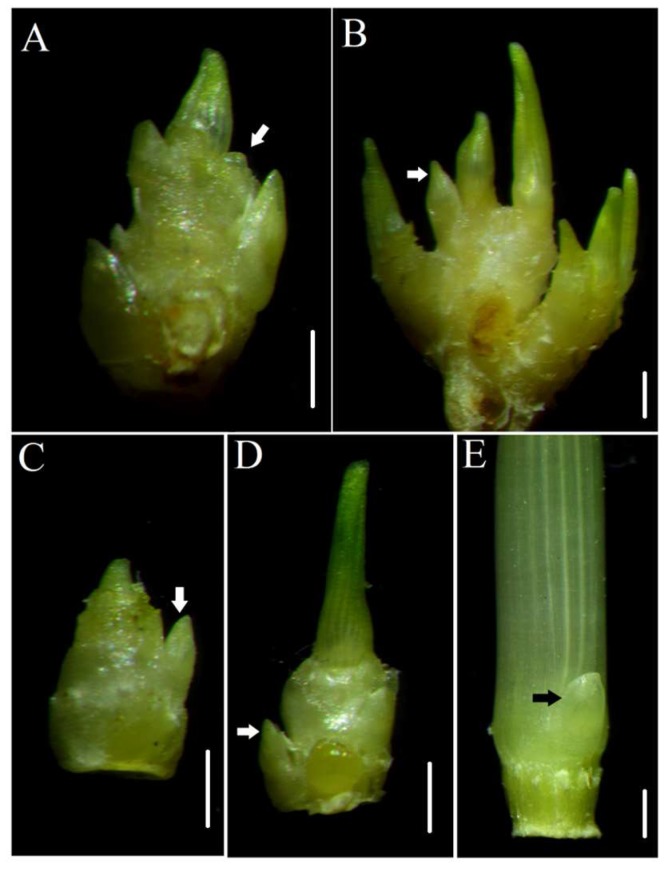
The tiller differentiation of the mutant *dmc* and WT. The tiller primordia of the WT (**A**) and mutant *dmc* (**C**) at the early tillering stage; the small tillers of the WT (**B**) and mutant *dmc* (**D**) at the middle tillering stage; and only one very small tiller at the basal node of *dmc* at the elongation stage (**E**). Arrow heads indicate the tiller primordia or small tillers, scale bar: 2 mm.

The mutant *dmc* was obtained from wheat cultivar “Guomai 301” treated with EMS. Mutant *dmc* basically does not tiller, and only has a main stem (Table 1, Figure 1A). Some individuals occasionally have a small tiller number. The plant height of the mutant *dmc* was significantly lower than that of the WT, i.e., 48.00 cm, which was 74.53% of the WT height (Table 1, Figure 1A). Both the spike length and the seed length of the mutant *dmc* were shorter than those of the WT (Table 1, Figure 1B–D). The diameters of the internodes of the mutant *dmc* were reduced (Figure 1E). The internode number of the mutant *dmc* was four; however, that of the WT was five (Table 1, Figure 1F). Additionally, we continuously observed and compared the tillers of the mutant *dmc* and WT from the early tillering stage. The mutant *dmc* had no or one tiller at the early tillering stage (Figure 2C), and it had no more tillers during the middle tillering period. Its tillering was significantly inhibited (Figure 2D). Generally, the mutant *dmc* had only one main stem at the jointing stage (Figure 2E). The tiller primordia (Figure S1) were used as samples for transcriptomic analysis in this study. Tiller primordium samples were dissected at the three-leaf stage to four-leaf stage. Two super bulk samples of the mutant *dmc* (T1, T2, and T3) and WT (T4, T5, and T6) with three biological replicates were prepared. Each bulk sample included more than ten independent individuals.

### 2.2. Genetic Diversity between the WT and Mutant dmc

To validate whether the *dmc* was derived from random open pollination in the field, Polymerase Chain Reaction (PCR) amplification of the WT and mutant *dmc* were carried out with 431 primer pairs of wheat Simple Sequence Repeats (SSR) markers evenly distributed on 21 wheat chromosomes. There was no polymorphic SSR locus between WT and mutant *dmc* (Figure S2), which demonstrated that their genetic backgrounds were highly consistent. This demonstrated that *dmc* was a real mutant derived from WT.

### 2.3. RNA Sequencing Data 

Six libraries were analyzed using RNA sequencing (mutant *dmc*: T1, T2, T3; WT: T4, T5, T6). We obtained a total of 68.8 Gb clean bases and about 463 million reads (single-end reads) (Table S1). The GC contents of the six libraries were 55.84–56.85%, and the average Q30 percentage was 89.25% (Table S1). Most transcripts were 100 to 300 bp in length (Figure S3), and the biological replicates were highly consistent (Figure S4). The reads were compared with the *T. aestivum* reference genome. There were 374 million (80.74%) reads mapped to the reference genome (Table S2). The average percentage of unique mapped reads was more than 71% (Table S2). It was clear that a high-quality transcriptome data set was obtained. 

### 2.4. Annotation and Functional Classification of the Unigenes

In total, 113,619 unique genes (unigenes) were obtained from the six libraries, and 109,685 unigenes were annotated by BLAST in several databases (Table S3). Furthermore, 10,080 new genes among the 113,619 unigenes were obtained, and 7075 new genes were annotated (Table S4). Functional classification in GO showed that the DEGs were classified into cellular component, molecular function, biological process, and many subcategories. Within the cellular component, molecular function and biological process categories, the most represented DEGs were classified as “cell”, “organelle” and “cell part”, “catalytic activity”, “binding”; “metabolic process”, “cellular process” and “single-organism process” (Figure S5).

According to homology unigene distribution of various species in the Nr database (Figure S6), the order from highest to lowest abundance was *Aegilops tauschii*, *H. vulgare*, *Triticum urartu*, *T. aestivum*, *Brachypodium distachyon*, *O. sativa*, *Z. mays*, *Setaria italica* and *S. bicolor*. 

### 2.5. DEGs between the WT and Mutant dmc 

**Figure 3:**
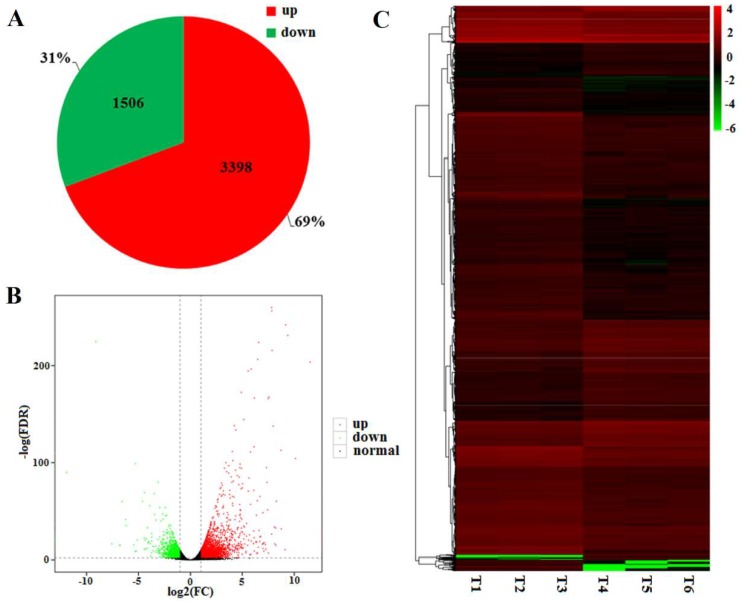
Expression level of DEGs in the mutant *dmc* compared to WT. (**A**) Pie chart of DEGs; (**B**) volcano plots of DEGs. The red and green dots represent genes expressed at high and low levels, respectively, in the mutant *dmc*; FC: Fold Change; FDR; false discovery rate; (**C**) Heatmap of DEGs. T1, T2, T3: mutant *dmc*; T4, T5, T6: WT. The color scale indicates the Log_2_ FPKM values.

To investigate the gene expression profile variation, a total of 4904 differentially expressed genes (DEGs) between the WT and mutant *dmc* were identified. Among them, 1506 were expressed at a low level, and 3398 were highly expressed in *dmc* compared to the WT (Figure 3A). The expression levels of DEGs in *dmc* and WT are shown as volcano plots (Figure 3B). The expression patterns of DEGs were hierarchically clustered (Figure 3C). The result showed there were significant DEGs, which may be the major genes related to wheat tillering. 

In order to further explore the key genes, significant DEGs (Log_2_FC ≥3 or ≤−2) were screened between the mutant *dmc* and WT (Table S5, Table S6). These DEGs were classified into several groups, such as transcription factor, signal transduction mechanism, and carbohydrate metabolism, etc. Chloroplastic d-3-phosphoglycerate dehydrogenase 2 was the most significant highly expressed gene in the mutant *dmc;* its Log_2_FC value was 11.43. Histone H2B.1 was the gene that was most significantly expressed at a low level in the mutant *dmc;* its Log_2_FC value was 12.28.

### 2.6. Functional Classification of the DEGs in GO

**Figure 4:**
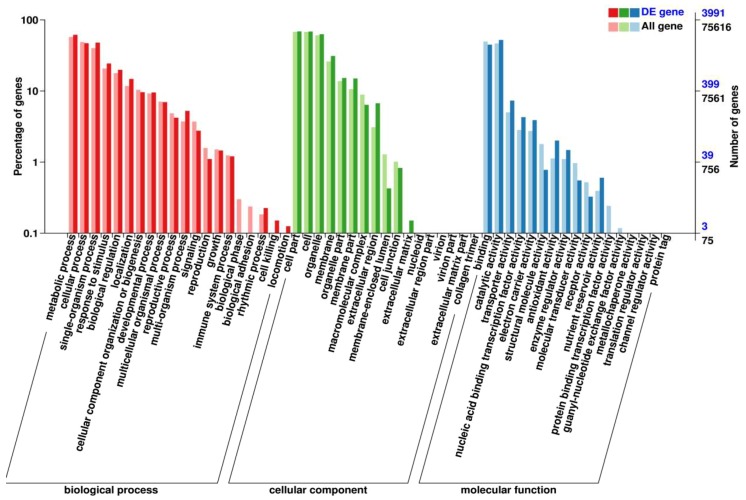
Functional classification of DEGs in the GO database.

To further explore wheat tiller–related biological pathways or processes, 3991 DEGs were classified into 54 subcategories in the GO database (Figure 4). According to the percentage of DEGs in all genes, the significant subcategories were biological phase (two DEGs), biological adhesion (three DEGs) (belonging to biological process), the membrane part (599 DEGs), the extracellular region (268 DEGs) and membrane-enclosed lumen (17 DEGs) (belonging to the cellular component), structural molecule activity (31 DEGs), molecular transducer activity (24 DEGs), receptor (13 DEGs), and protein binding transcription factor (two DEGs) (belonging to molecular function). 

### 2.7. Pathway Mapping of the DEGs in KEGG

**Figure 5:**
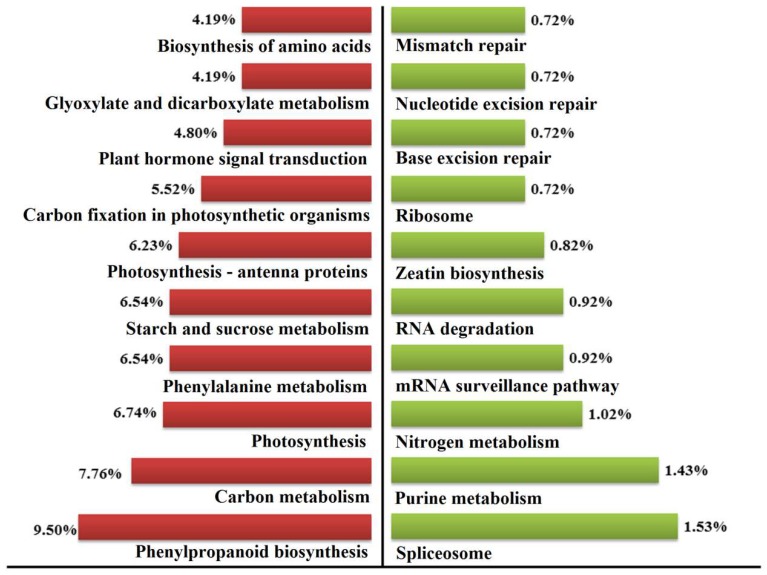
The top ten enhanced and suppressed pathways in the mutant *dmc* compared to the WT. (**Left**) The enhanced pathways; (**Right**) the suppressed pathways. Percentage: The ratio of the number of DEGs annotated to one pathway to the number of DEGs annotated to all pathways.

A total of 979 DEGs were assigned to 112 pathways in KEGG (Table S7). The top ten enriched pathways of enhanced and suppressed DEGs were obtained (Figure 5). The top ten enhanced pathways were phenylpropanoid biosynthesis (ko00940), carbon metabolism (ko01200), photosynthesis (ko00195), starch and sucrose metabolism (ko00500), phenylalanine metabolism (ko00360), photosynthesis-antenna proteins (ko00196), carbon fixation in photosynthetic organisms (ko00710), plant hormone signal transduction (ko04075), biosynthesis of amino acids (ko01230), and glyoxylate and dicarboxylate metabolism (ko000630). In contrast, the top ten suppressed pathways were spliceosome (ko03040), purine metabolism (ko00230), nitrogen metabolism (ko00910), mRNA surveillance pathway (ko03015), RNA degradation (ko03018), zeatin biosynthesis (ko00908), ribosome (ko03010), base excision repair (ko03420), mismatch repair (ko03430), and nucleotide excision repair (ko03420). 

**Figure 6:**
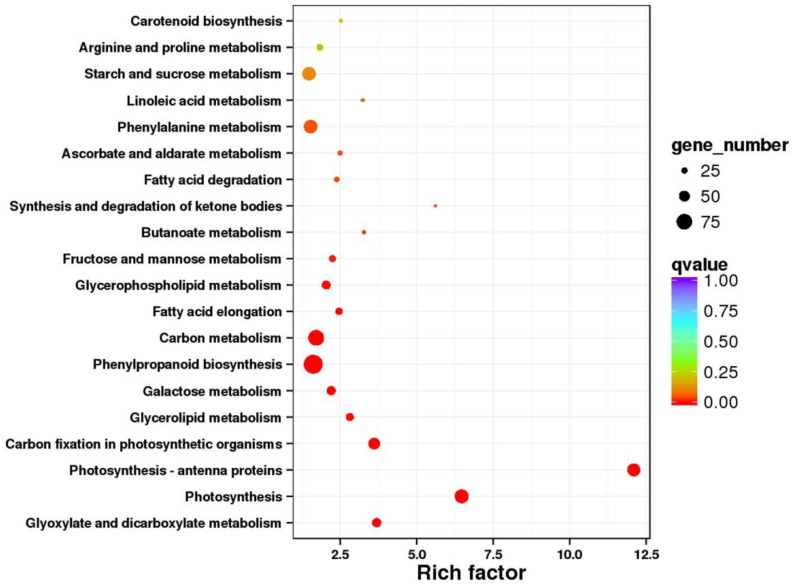
Significance analysis of the enriched pathways. Rich factor: the percentage of A/B. A: The percentage of DEGs annotated to a pathway. B: The percentage of unigenes annotated to a pathway. *Q* value: corrective *p* value.

The enriched pathways were analyzed by a significance test, and twenty significantly enriched (*Q* value < 0.2) pathways were obtained in the mutant *dmc* (Figure 6, Table S8), such as photosynthesis-antenna proteins (ko00196), carbon metabolism (ko01200), carbon fixation in photosynthetic organisms (ko00710), fatty acid elongation (ko00062), butanoate metabolism (ko00650), and phenylpropanoid biosynthesis (ko00940).

### 2.8. The DEGs Involved in Phytohormone Metabolisms in dmc

**Figure 7:**
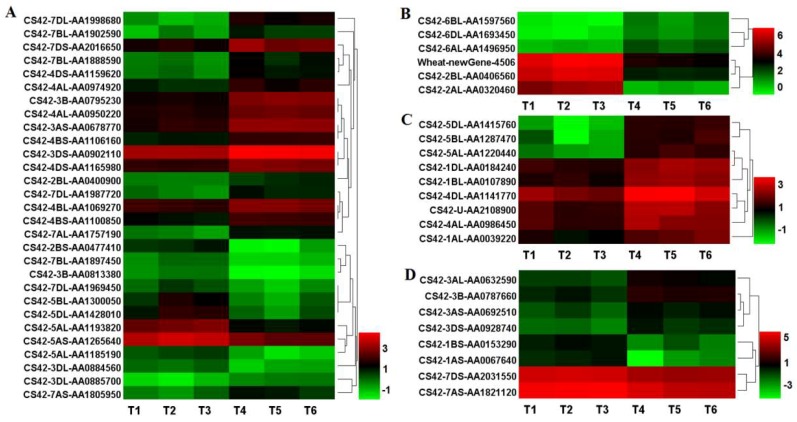
Heatmap of the DEGs involved in plant hormone metabolisms. (**A**) The DEGs in auxin metabolism; (**B**) the DEGs in abscisic acid metabolism; (**C**) the DEGs in cytokinin metabolism; and (**D**) the DEGs in gibberellin metabolism. T1, T2, T3: mutant *dmc*; T4, T5, T6: WT The color scale indicates the Log_2_ FPKM values.

The DEGs involved in plant hormone metabolisms were enriched in the mutant *dmc*, and a total of 83 DEGs were obtained (Figure 7, Table S9). Among them, 52 DEGs were expressed at a low level, and 31 DEGs were highly expressed in the mutant *dmc* compared to the WT. The DEGs of cytokinin hydroxylase, cytokinin dehydrogenase and cytokinin phosphoribohydrolase were all expressed at a low level (Figure 7C, Table S9); three DEGs of abscisic acid 8′-hydroxylase and abscisic stress-response protein were expressed at a high or low level (Figure 7B, Table S9). Two DEGs of gibberellin 20 oxidase and gibberellin 2-beta-dioxygenase were expressed at a low level (Figure 7D, Table S9); 19 DEGs involved in auxin metabolism were expressed at a low level (Figure 7A, Table S9), 17 DEGs involved in ethylene metabolism were expressed at a low level in the mutant *dmc* compared to the WT (Table S9). Abscisic stress-responding protein 3 (AA0320460) and auxin-induced protein X15 (AA1998680) were the DEGs that were most significantly expressed at high and low levels, respectively, in the mutant *dmc* compared to the WT (Table S9).

### 2.9. The DEGs Related to Carbohydrate Metabolism in the Mutant dmc

**Figure 8:**
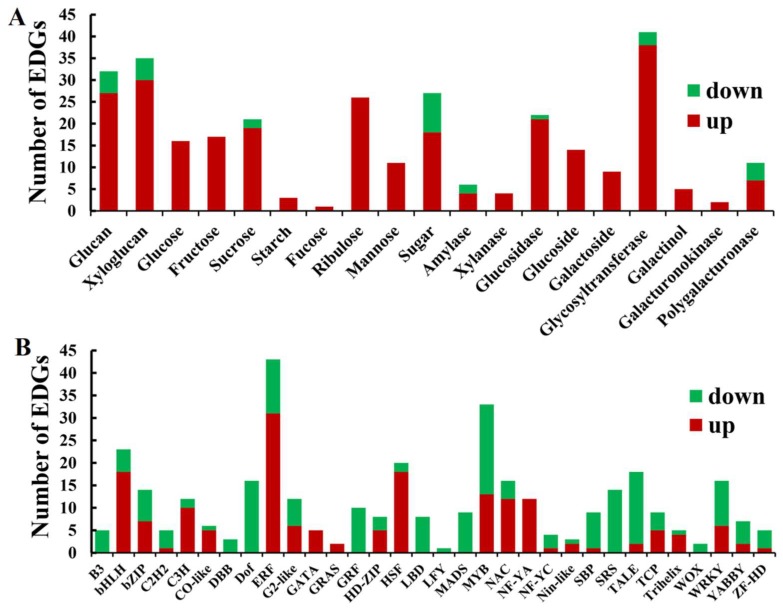
A bar chart of the DEGs involved in carbohydrate metabolisms and the transcription factors. (**A**) Classification of the DEGs involved in carbohydrate metabolisms. *x*-axis: the subcategories of carbohydrate metabolisms, *y*-axis: the number of DEGs in each subcategory; (**B**) Classification of the differentially-expressed transcription factors. *x*-axis: The subfamilies of transcription factors, *y*-axis: the number of DEGs in each family of transcription factors. Red: highly-expressed DEGs; green: DEGs expressed at a low level.

A total of 303 DEGs related to carbohydrate metabolism were obtained and they were divided into 19 groups (Figure 8A, Table S10). Among these, the DEGs related to the metabolism of glucose, fructose, starch, fucose, ribulose, mannose, xlanase, glucoside, galactoside, galactinol and galacturonokinase were all highly expressed in *dmc* (Figure 8A, Table S10). The DEGs related to metabolisms of glucan, xyloglucan, sucrose, sugar, amylase, glucosidase, glycosyltransferase, and polygalacturonase were simultaneously expressed at high or low levels in *dmc*. Furthermore, the percentages of DEGs expressed at low levels were lower than those of the highly-expressed DEGs in all carbohydrate metabolisms (Figure 8A, Table S10).

### 2.10. Transcription Factor Type DEGs in the Mutant dmc

A total of 35 significant DEGs of transcription factors (Log_2_FC ≥3 or ≤−2) were obtained in *dmc* (Figure 8B, Table S5, Table S6), and they belonged to zinc finger protein, heat stress transcription factor, NAC domain-containing protein, WRKY transcription factor, ethylene-responsive transcription factor and MADS-box protein families, etc. Among these DEGs regulated by transcription factors (Figure 8B, Table S11), an SPX domain-containing membrane protein Os02g45520 (AA1610920) was the most significantly highly expressed, and probable WRKY transcription factor 12 (AA0534130) was the DEG that was most significantly expressed at a low level in the mutant *dmc* compared to the WT. The DEGs belonging to families B3, DBB, Dof, GRF, LBD, LFY, MADS, SRS, and WOX were only expressed at low levels; the DEGs belonging to families GATA, GRA, and NF-YC were only expressed at high levels in the mutant *dmc* compared to the WT (Figure 8B, Table S11). The most enriched transcription factor family was ERF, followed by MYB, bHLH and HSF (Figure 8B, Table S11). 

### 2.11. The DEGs Related to Signaling Processes

A total of 390 DEGs related to signal transduction were obtained (Table S12). Among these DEGs, signal transduction NRT1/PTR family protein genes occupied the largest percentage (8.72%), followed by F-box protein (5.13%), E3 ubiquitin-protein ligase (5.13%), pentatricopeptide repeat-containing protein (4.62%), CBL-interacting protein kinase (4.36%) and cysteine-rich receptor-like protein kinase (3.85%) (Table S12). The growth regulating factor, leucine-rich repeat extensin-like protein, phytosulfokine receptor, protein G1-like, protein reveille, protein short internodes and protein SHI related sequence genes were all expressed at low levels. Ankyrin repeat domain-containing protein, basic 7S globulin 2 low molecular weight subunit, CBL-interacting protein kinase 14, NAC domain-containing protein, and pectinesterase were almost always highly expressed (Table S12).

### 2.12. Expression Profiles of Twelve Genes in Wheat Tiller Primordia

**Figure 9:**
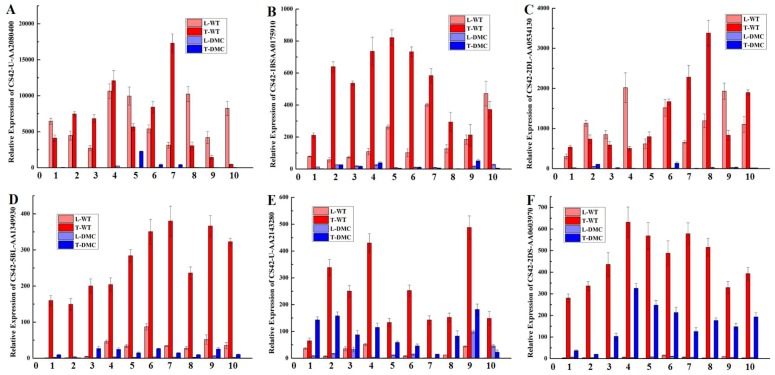

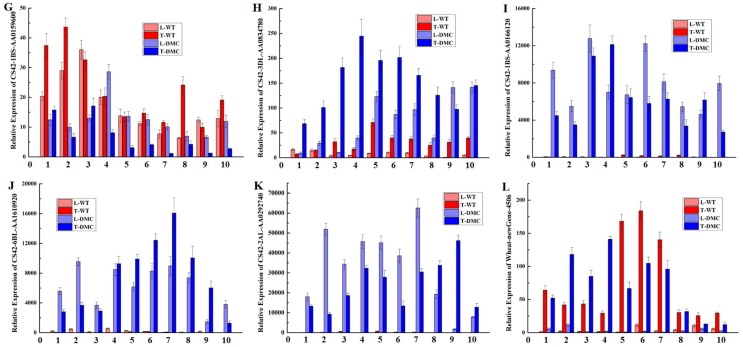
Temporal expression profiles of the twelve genes in wheat tiller primordia and leaves. (**A**) Histone H2B.1 gene (CS42-U-AA2080400); (**B**) PGR5-like protein 1A gene (CS42-1BS-AA0175910); (**C**) WRKY transcription factor 12 gene (CS42-2DL-AA0534130); (**D**) ATP-dependent zinc metalloprotease FTSH 5 gene (CS42-5BL-AA1349930); (**E**) BOI-related E3 ubiquitin-protein ligase 3 gene (CS42-U-AA2143280); (**F**) Acid phosphatase 1 gene (CS42-2DS-AA0603970); (**G**) Bidirectional sugar transporter SWEET3a gene (CS42-1BS-AA0159600); (**H**) Arginine decarboxylase gene (CS42-3DL-AA0834780); (**I**) F-box/FBD/LRR-repeat protein At5g22670 gene (CS42-1BS-AA0166120); (**J**) SPX domain-containing membrane protein Os02g45520 gene (CS42-6BL-AA1610920); (**K**) GDSL esterase/lipase At1g28600 gene (CS42-2AL-AA0292740); (**L**) Abscisic stress-ripening protein 1 gene (Wheat-newGene-4506). T: tiller primordia; L: leaves; WT: Guomai 301; DMC: mutant *dmc*; Error bars indicate the standard deviation. *x*-axis: 10 time points of the sample preparation; the intervals were seven days from 23 December 2016 to 24 February 2017. All qRT-PCR reactions were replicated three times.

To demonstrate the reliability of the sequencing results, we selected twelve of the significant DEGs to perform real-time qRT-PCR. Linear regression analysis showed that the results of the transcriptome analysis were reliable (Figure 9, Figure S7).

A histone H2B.1 gene (CS42-U-AA2080400, Figure 9A), a PGR5-like protein 1A gene (CS42-1BS-AA0175910, Figure 9B) and a WRKY transcription factor 12 gene (CS42-2DL-AA0534130, Figure 9C) were significantly suppressed in tiller primordia of *dmc*, and these three genes were also significantly suppressed in the leaves of *dmc* compared to the WT. Mutation significantly suppressed the expression of an ATP-dependent zinc metalloprotease FTSH 5 gene (CS42-5BL-AA1349930, Figure 9D), a BOI-related E3 ubiquitin-protein ligase 3 gene (CS42-U-AA2143280, Figure 9E), an acid phosphatase 1 gene (CS42-2DS-AA0603970, Figure 9F) and a bidirectional sugar transporter SWEET3a gene (CS42-1BS-AA0159600, Figure 9G) in tiller primordia of *dmc*; these four genes were specifically expressed in tiller primordia compared to leaves except the bidirectional sugar transporter SWEET3a gene (CS42-1BS-AA0159600, Figure 9G). The expression of an arginine decarboxylase gene (CS42-3DL-AA0834780, Figure 9H); a putative F-box/FBD/LRR-repeat protein At5g22670 gene (CS42-1BS-AA0166120, Figure 9I); a SPX domain-containing membrane protein Os02g45520 gene (CS42-6BL-AA1610920, Figure 9J); and a GDSL esterase/lipase At1g28600 gene (CS42-2AL-AA0292740, Figure 9K) were significantly activated in tiller primordia of *dmc* compared to WT, and their expression profiles in leaves of *dmc* were also similar. The expression of an abscisic stress-response protein 1 gene (Wheat-newGene-4506, Figure 9L) was irregular. The twelve genes were either only just expressed or completely suppressed in tiller primordia of *dmc*. The results suggested that these genes played important roles in the tiller development of wheat.

## 3. Discussion

### 3.1. Lack of Vitality in the Mutant dmc 

The morphology of the mutant *dmc* was dwarfish, with yellowish cotyledons and almost no tillers, which demonstrated its lack of vitality. The phenotype of the mutant *dmc* was novel. In wheat, the leaves of the tiller inhibition mutants *tin3* and *ftin* were much darker than the WT plants [1,4]; however, the leaves of the mutant *dmc* were yellowish. Both spikes and seed were much larger in the mutant *tin3* [1] compared with the mutant *dmc*. Normally, there is a highly negative correlation between tiller number and plant height in rice and wheat [6]. In wheat, *NAUH167*, a high-tillering mutant was dwarfish compared to the WT [6], the plant height of the tiller inhibition mutant *ftin* was slightly higher than that of the WT [4], the phenotype of the mutant *dmc* was not the same as *NAUH167* or *ftin*, and the mutant *dmc* was not only dwarfish but also had almost no tillers. The mutant *dmc* had fewer tiller primordia. Most of the primordia could not grow or stopped developing, which resulted in no tillers. Though most DEGs related to photosynthesis were highly expressed in *dmc*, this occurred in contrast to its lower vitality and biomass.

### 3.2. Auxin and Cytokinin Metabolisms Were Suppressed in dmc

Phytohormones play essential roles in plant growth and development throughout the vegetative to reproductive stages [40,41,42,43]. Tillering is a key developmental event in wheat, which needs a balanced phytohormone metabolism to maintain normal tillering [44,45,46]. 

In Arabidopsis, auxin can inhibit bud outgrowth in the highly branched shoot mutant *axr1*, and the effect of auxin on the wild type is more obvious [47]. In rice, a knock-down mutant of *OsIAA6* produces more tillers due to the regulation of the auxin transporter OsPIN1 [48]. However, a further study found that auxin does not enter the lateral buds [49,50], and this phenomenon can be supported by cytokinin and strigolactone [50,51]. In Arabidopsis, cytokinin acts to overcome auxin-mediated apical dominance, allowing buds to evade apical dominance [52]. In this study, we obtained 29 DEGs related to auxin metabolism, and 19 DEGs were expressed at a low level (Figure 6, Table S9): the level of auxin in the mutant *dmc* might be low; however, this might not be consistent with the premise that auxin inhibits bud outgrowth. The KEGG pathway of zeatin biosynthesis (ko00908; Figure S8) was suppressed, and the suppressed differentially expressed zeatin biosynthesis genes were adenylate isopentenyltransferase 1 (chloroplastic), three cytokinin hydroxylases, and three cytokinin dehydrogenases (Figure S8, Table S9). Furthermore, the plant hormone signal transduction genes (ko04075; Figure S9) in KEGG showed that the expression of zeatin biosynthesis can affect cytokinin biosynthesis: the expression of downstream signal transduction components and the two-component response regulator (*ARR*) were down-regulated (Figure S9). This result is in agreement with a previous study on rice, which indicated that a high level of cytokinin can increase the tiller number [53].

Interestingly, overexpressing gibberellin 2-oxidases (*GA2ox*) can produce more tillers in rice [54]. Gibberellin localization in vascular tissue is required to control auxin-dependent bud outgrowth in hybrid aspen (*Pterocarya stenoptera*) [55]. Gibberellin is also required for cytokinin-mediated axillary bud outgrowth in *Jatropha curcas* [56]. In this study, two DEGs encoding gibberellin 20 oxidase 2, and DEGs encoding gibberellin 2-beta-dioxygenase 1 and gibberellin 3-beta-dioxygenase 2-1, which were expressed at low levels, were obtained (Figure 6, Table S9), and the possible high level of gibberellin could be consistent with the mutant *dmc* not tillering. The hormone regulation of wheat tillers is very complex, and further research is needed in the future.

The plant height of the mutant *dmc* was decreased significantly. It was about 74.53% of the WT height (Figure 1A). Many plant hormones can promote plant growth, like auxin [42], gibberellin [57,58], and cytokinin [59]. The suppressed expression of genes involved in auxin, zeatin and cytokinin metabolisms (Figure S9) may be the key causes accounting for dwarfism of the mutant *dmc*.

### 3.3. Carbohydrate Metabolism and Phenylpropanoid Biosynthesis Were Active in dmc

Many studies have demonstrated that sugar can regulate bud growth [60,61,62]. In wheat, the phenotype of the tiller inhibition mutant (*tin*) was associated with precocious internode elongation; further study showed that the mutant *tin* transferred sucrose away from buds to elongating internodes [61]. In pea (*Pisum sativum*), total sucrose levels were significantly increased in node 2 buds after decapitation; the axillary buds were rapidly released in intact plants supplemented with external sucrose [60]. Sugar also affects bud outgrowth in rose (*Rosa* sp.) [62,63]. In this study, the enriched pathways of starch and sucrose metabolism (ko00500) in KEGG were significantly activated (Figure 5, Figure 8A). Two DEGs related to sucrose synthase were expressed at a low level, and nine DEGs related to sucrose 1-fructosyltransferase, which were expressed at a high level, were obtained (Figure 7, Table S10). The potentially higher activities of the corresponding enzymes may decrease the content of small molecular carbohydrates, which may inhibit wheat tillering. 

Interestingly, phenylpropanoid biosynthesis (ko00940; Figure S10) was the most enhanced pathway in KEGG, which regulates the biosyntheses of different lignins such as syringyl, guaiacyl, *p*-hydroxy-phenyl, and 5-hydroxy-guaiacyl lignins. A total of 93 annotated DEGs belonged to the phenylpropanoid biosynthesis (Figure S10), and among them 56 were various peroxidases (Table S10) which likely suppressed the biosyntheses of lignins, and the concentration of lignins in the mutant might be low. This result might be consistent with the dwarf phenotype of the mutant *dmc*. 1-aminocyclopropane-1-carboxylic acid oxidase (ACCO) catalyzes the final step of ethylene biosynthesis. In this study, seven DEGs of ACCO and seven DEGs of ACCO homologs were obtained, and they might affect the biosynthesis of ethylene in *dmc*.

### 3.4. Some Transcription Factors Are Suppressed in dmc

Transcription factors (TFs) play essential roles in plant leaf [64], flower [65], and branch [66] growth and development.

In maize (*Zea mays*), the Knotted1-like homeobox (*KNOX*) TFs up-regulates *GA2ox1* [67]. In this study, four DEGs of knotted-1-like 1, three knotted-1-like 12 and three *KNOX3* (TALE family) were obtained, and they were all expressed at a low level, and these might down-regulate the expression of *GA2ox1* and inhibit tillering in the mutant *dmc* (Figure 8, Table S11). This observation is in agreement with the observation that overexpressing *GA2ox* produced more tillers in rice [54]. In rice, overexpression of growth-regulating factors *OsGRF3* and *OsGRF10* reduces tillers. The expression of *Oskn2*, one rice *KNOX* gene, was down-regulated by overexpression of *OsGRF3* [68]. In this study, one DEG homologous to TFs *GRF2*, *GRF10*, and *GRF12*; two DEGs homologous to *GRF1* and *GRF5*; three DEGs homologous to *GRF9* were obtained (GRF family), however, they were all expressed at a low level in *dmc* (Figure 8, Table S11). The relationship between *GRFs* expressed at a low level and the phenotype of the mutant *dmc* needs further study.

DWARF TILLER1, a WUSCHEL-related homeobox (*WOX*) TFs, is a positive regulator of tiller growth in rice [66]. In rice, the completely sterile and reduced tillering 1 mutant (*srt1*) was caused by a mutation in WUSCHEL (*OsWUS*, one member of the WOX gene family). The homeobox domain of SRT1 is essential for tillering [69]. In this study, two DEGs homologous to TFs *WOX 4* (WOX family) were expressed at a low level in *dmc* (Figure 8, Table S11), which might affect the tillering of *dmc*. Furthermore, The DEGs of TF families B3, DBB, Dof, GRF, LBD, LFY, MADS and SRS were only expressed at a low level (Figure 8, Table S11); they might affect the tillering of *dmc*.

### 3.5. Signal Transduction and Photosynthesis in dmc

Signal transduction is related to many pathways and metabolic processes. In Arabidopsis, short internodes (SHI) is a suppressor of GA responses [70]. In this study, six DEGs associated with short internodes, expressed at a low level, were obtained, and they might promote GA responses in the *dmc* mutant that does not produce tillers. The larger percentage of DEGs related to E3 ubiquitin-protein ligase, NRT1/PTR FAMILY, F-box protein, pentatricopeptide repeat-containing protein and E3 ubiquitin-protein ligase (Table S12) might also affect the tillering and plant growth of the mutant *dmc*.

Photosynthesis (ko00195; Figure S11) was the third most enhanced pathway in the mutant *dmc* (Figure 4), and photosystem I, photosystem II, cytochrome b6/f complex, photosynthethic electron transport and F-type ATPase related to photosynthesis were all highly expressed (Figure S11). 

The enhanced expression of the photosynthetic components did not correspond with the phenotype of the mutant *dmc*, which requires further study.

## 4. Materials and Methods 

### 4.1. Plant Materials and Growth Conditions 

The wheat cultivar ‘Guomai 301’ was bred in our laboratory. Wheat cultivar “Guomai 301” has medium tillers, but a high percentage of earbearing tillers. The tillering ability of the WT conformed to the requirements of high-yield wheat breeding, which ensures a relatively dense population (600–700 spikes per hectare) and large spikes with more grains (36.1–37.4 grains per spike). In October 2012, the seeds of the WT were treated with EMS and planted at the Shangqiu Experimental Farm, Henan Province, China (34°25′ N, 115°39′ E, 49 m a.s.l.). The mutant *dmc* was obtained from the M_2_ generation in 2013. Thereafter, to eliminate the background mutations, *dmc* plants were individually selected generation by generation; normal plants in the segregating line were also selected as the control (CK in line; WT) simultaneously. In 2016, the WT and *dmc* were planted at the Experimental Farm of Henan Agricultural University, Zhengzhou, Henan Province, China (34°51′ N, 113°35′ E, 95 m a.s.l.). The lines were sown in plots of 3.0 m in length and 2.0 m in width; the distance between rows was 0.25 m, and 20 seeds were planted in each row [71]. The samples for transcriptome sequencing analysis were prepared in 2017.

### 4.2. Trait Measurements, Morphology Observations and SSR Analysis

The tiller number, spike number, plant height, and internode number of the main stem, among other traits (Table 1), of *dmc* and the WT were observed and measured. Each sample was prepared by random selection of more than 20 individuals of *dmc* and the WT. The statistical tests were performed using Student’s *t*-test, and the variation was expressed as the standard deviation (SD).

The tillers of the mutant *dmc* and WT were observed from the early tillering stage with an inverted microscope (Olympus 3111286), and the images were captured by a camera (Nikon Coolpix 4500). The outer leaves and sheaths were removed to show the tiller primordia and very small tillers at the seedling stage with an anatomical needle. The large tillers at the adult plant stage were observed by the naked eye.

A set of microsatellite assays was applied to evaluate the genetic diversity among the WT and mutant *dmc*. The SSR primers (Appendix) employed in this study included those of GDM [72], WMC [73], etc. Genomic DNAs of the WT and mutant *dmc* were extracted from leaves with the CTAB method [74]. The PCR reactions were performed in 10 µL volumes containing 5 µL EasyTaq^®^ PCR SuperMix for PAGE (2×) (TransGen Biotech, Beijing, China), 1 µL primer mix (10 µM), 0.5 µL DNA (100 ng) and 3.5 µL ddH_2_O. The PCR parameters were: 94 °C for 5 min, then 33 cycles of 94 °C for 30 s, 50–60 °C (based on primer annealing temperature) for 30 s and 72 °C for 60 s, and a final elongation at 72 °C for 10 min. PCR products were separated in 8% non-denatured polyacrylamide gels (acrylamide:bisacrylamide = 19:1) at room temperature. Each sample (2 µL) was loaded and run in 1× TBE (90 mmol L–1 Tris-borate, 2 mmol L–1 of EDTA, pH 8.3) buffer at 90 W for 1 h, then visualized by silver staining [75].

### 4.3. RNA Extraction, Library Preparation and Sequencing

Tiller primordial samples (Figure S1) were dissected at the three-leaf stage to four-leaf stage. Two super bulk samples of the mutant *dmc* (T1, T2, and T3) and WT (T4, T5, and T6) with three biological replicates were prepared. Each bulk sample included more than ten independent individuals. 

RNA was extracted using Trizol reagent (TransGen Biotech, Beijing, China) according to the manufacturer’s protocol. RNA concentration was measured using a NanoDrop 2000 (NanoDrop Technologies, Wilmington, DE, USA). RNA integrity was assessed using the RNA Nano 6000 Assay Kit on the Agilent Bioanalyzer 2100 system (Agilent Technologies, Santa Clara, CA, USA).

The RNA bulk samples were prepared with 1 μg RNA per individual. Sequencing libraries were constructed using NEBNext UltraTM RNA Library Prep Kit for Illumina (NEB, Ipswich, MA, USA) following the manufacturer’s recommendations. The preferential cDNA fragments were 240 bp in length. The correlation among biological replicates was analyzed using Pearson’s Correlation Coefficient (r) [76]. The six cDNA libraries were sequenced with Illumina HiSeq Xten from Biomarker Biotechnology Corporation (Beijing, China). 

### 4.4. Transcriptome Analyses

The clean reads were compared with the *T. aestivum* reference genome [77] using TopHat2 [78]. The unigenes were annotated by BLAST [79] in Nr (NCBI non-redundant protein sequences), Nt (NCBI non-redundant nucleotide sequences), Pfam (protein family), KOG/COG (Clusters of Orthologous Groups of proteins), Swiss-Prot (a manually annotated and reviewed protein sequence database), KO (KEGG Ortholog database), and GO (Gene Ontology) databases. To explore new transcripts and genes in the mutant *dmc*, the reads were assembled using Cufflinks software [80] based on the reference genome sequence, and the transcriptional intervals that had not been annotated were searched for and compared with the original genome annotation information. The KEGG Orthology of the new genes was analyzed using KOBAS2.0 [81], and the amino acid sequences of the new genes were predicted; finally, the new genes were annotated using HMMER [82], referring to the numerous databases. The standard gene expression levels were expressed as fragments per kilobase of transcript per million fragments mapped (FPKM) [83]. Differentially-expressed genes (DEGs) were identified by DEseq [84] with a false discovery rate (FDR) <0.01 and a fold change value (FC) ≥2. 

### 4.5. qRT-PCR 

The tiller primordia and leaf samples of the mutant *dmc* and guomai 301 were prepared at 10 time points for real-time qRT-PCR; the intervals of the 10 time points were seven days from 23 December 2016 to 24 February 2017. Twelve DEGs were selected to test and verify the sequencing data by qRT-PCR. The primers were designed using Primer Premier 5.0 (Table S13). The wheat *actin* gene was used as an internal control gene. The qRT-PCR reactions were performed in 20 µL volumes containing 10 µL TransStart^®^ Top Green Qpcr SuperMix (2×) (TransGen Biotech, Beijing, China), 2 µL primer mix (10 µM), 1 µL cDNA (50 ng) and 7 µL ddH_2_O. The PCR parameters were: 94 °C for 30 s, then 42 cycles of 94 °C for 5 s, 60 °C for 30 s. All qRT-PCR reactions were replicated three times. The gene expression levels were calculated according to the 2^−ΔΔ*C*t^ method [85]. 

### 4.6. Ethical Approval

This article does not contain any studies with human participants or animals performed by any of the authors.

Wheat (*Triticum aestivum* L.) plants were used in this study. The wheat cultivar ‘Guomai 301’ was bred in our laboratory.

The wheat mutant *dmc* was selected from EMS treated Guomai 301 in our laboratory.

## 5. Conclusions

Transcriptomes of the tiller primordia from the wheat non-tiller mutant *dmc* and WT Guomai 301 were integratively analyzed. We identified a set of genes related to wheat tiller differentiation. Sixty-nine percent of significant DEGs (FC ≥ 2) were highly expressed and 31% were expressed at a low level in *dmc*. Phenylpropanoid biosynthesis (ko00940; Figure S10) had the most highly expressed DEGs (9.50%). Carbohydrate-related metabolisms consisted of the largest highly-expressed DEG group, including photosynthesis (6.74%), starch and sucrose metabolism (6.54%), photosynthesis-antenna proteins (6.23%), carbon fixation in photosynthetic organisms (5.52%), carbon metabolism (7.76%), and galactose metabolism (4.09%). The majority of genes that were expressed at a low level belonged to the classes of DNA replication, transcription, and translation, as well as zeatin synthesis. Functional model analysis indicated that variations in carbohydrate metabolism, phytohormones and transcription factors were the major causes of non-tillering in *dmc*. Synthesis and lower expression of genes related to protein synthesis, auxin, zeatin and cytokinine; syntheses enhanced the expression of genes involved in the biosynthesis of abscisic acid, gibberellins, and ethylene syntheses, consistent with the phenotype of *dmc*. The expression profiles of TF homologs, knotted-1-like 1, homeobox protein knotted-1-like 12 and *KNOX3*, and *WOX 4* were also consistent with the phenotype of *dmc*. Other issues need further study, such as the relationship between phenylpropanoid biosynthesis, photosynthesis, variations of different phytohormone concentrations and the phenotype of *dmc*.

## Figures and Tables

**Table 1:** Comparison of agronomic traits between the WT and mutant *dmc*.

Traits	WT	*dmc*
Plant height/cm	64.45 ± 3.28	48.00 ± 2.63 **
Spike length/cm	10.47 ± 0.53	6.48 ± 0.90 **
Internode number of main stem	5.00 ± 0	4.18 ± 0.51 **
The top first internode length of the main stem/cm	21.74 ± 2.28	19.46 ± 2.37
The top second internode length of the main stem/cm	13.98 ± 0.27	11.21 ± 1.55 *
The top third internode length of the main stem/cm	9.42 ± 0.74	6.48 ± 0.34 **
The top fourth internode length of the main stem/cm	5.78 ± 0.29	4.24 ± 0.22 **
The top fifth internode length of the main stem/cm	3.19 ± 0.31	-
Tiller number	21.73 ± 2.20	1.11 ± 0.3 **
Spike number	16.64 ± 0.92	1.06 ± 0.32 **
Spikelet number on the main stem	21.73 ± 1.19	14.85 ± 2.09 **
Seed number per spike	64.10 ± 5.36	34.16 ± 4.28 **
1000-grain weight/g	44.17 ± 4.59	35.41 ± 4.34 **
Heading stage/week	27	28
Anthesis stage/week	28	29
Maturity stage/week	32	33

* *p* < 0.05; ** *p* < 0.01.

## Supplementary Materials

Supplementary materials can be found at http://www.mdpi.com/1422-0067/19/5/1324/s1.
