# Supplementary figures and images for: Quantitative Changes in the Transcription of Phytohormone-Related Genes: Some Transcription Factors Are Major Causes of the Wheat Mutant dmc Not Tillering
Source: Int J Mol Sci. 2018 Apr 29;19(5):1324. doi: 10.3390/ijms19051324 (PMC5983577; doi:10.3390/ijms19051324)

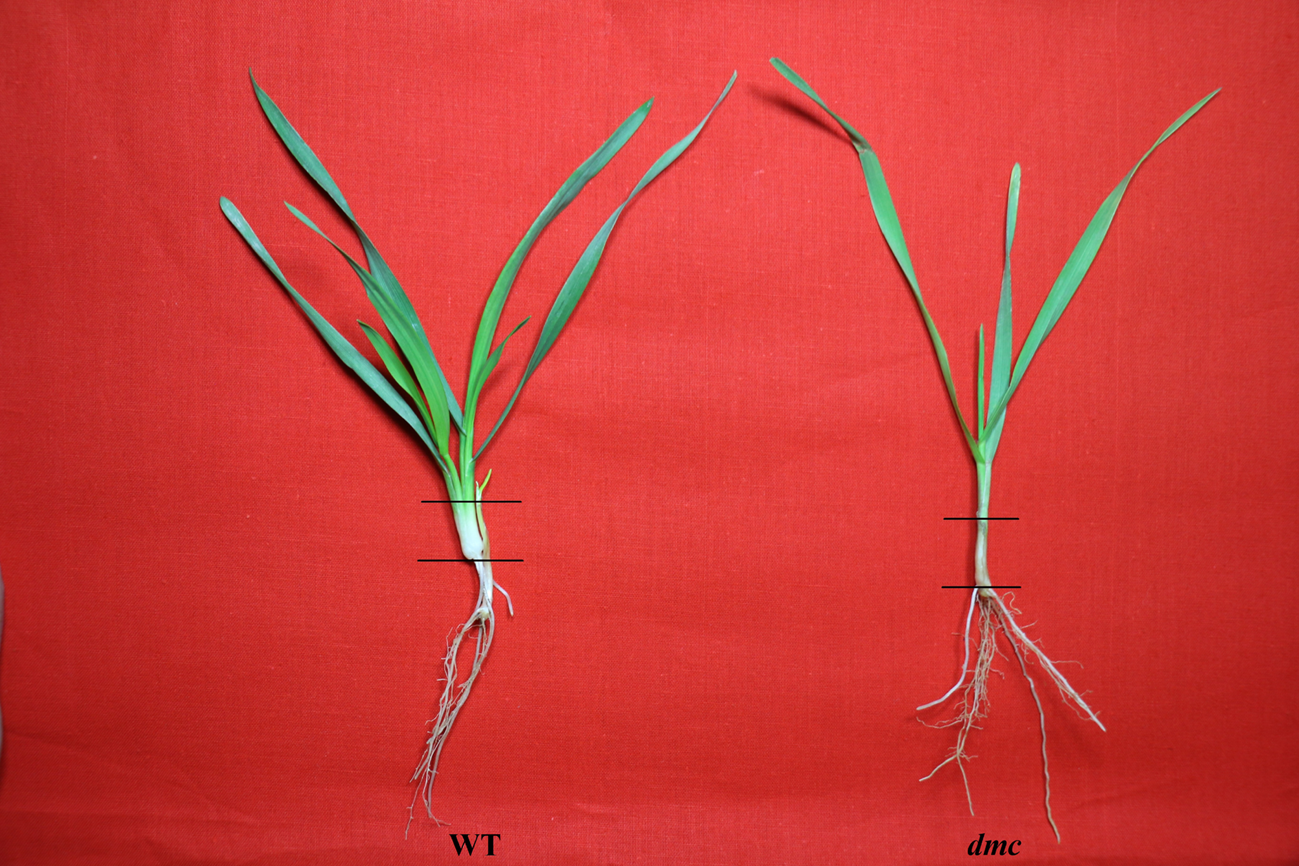

Supplement: Supplementary file 1 [file ijms-19-01324-s001.zip › Figure S1.tif]

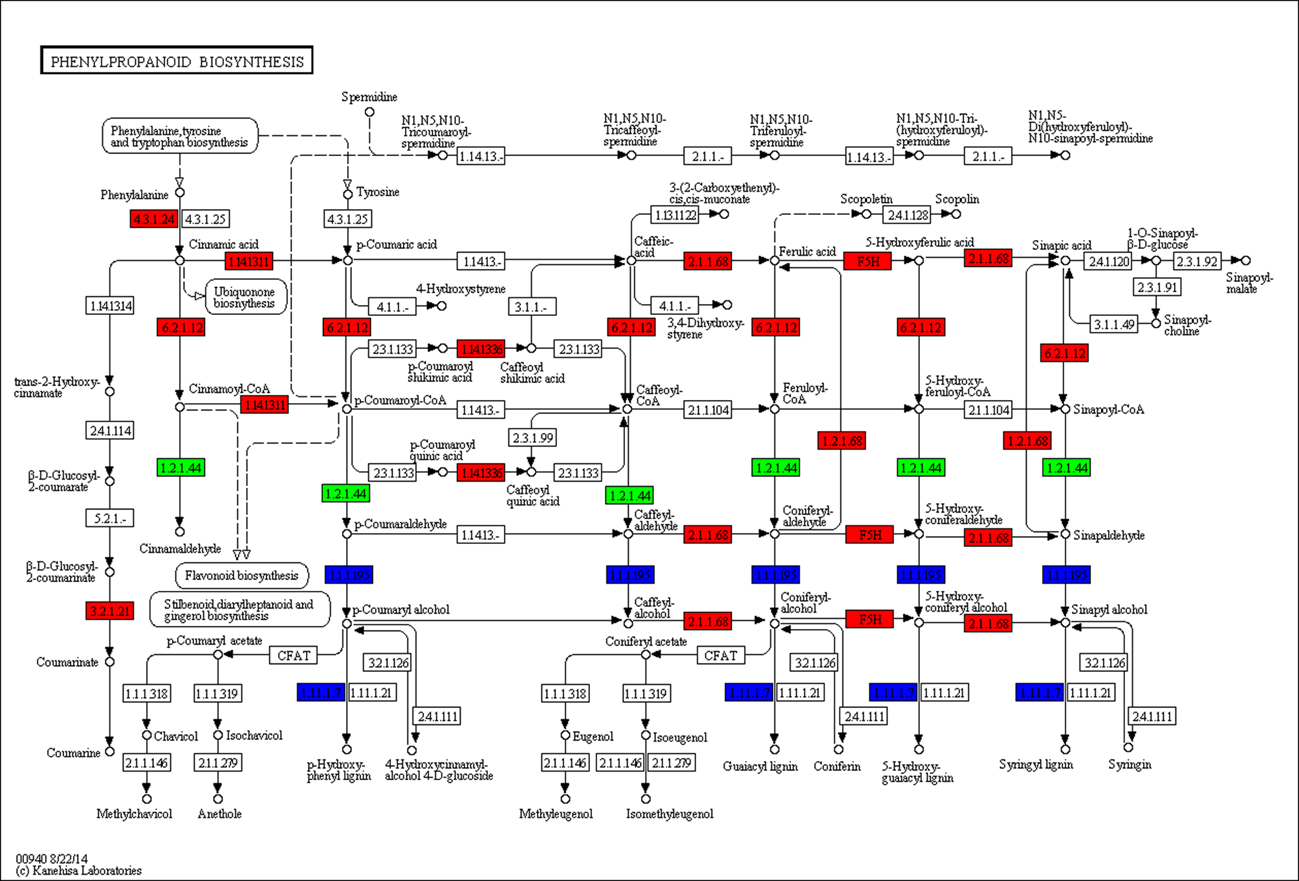

Supplement: Supplementary file 1 [file ijms-19-01324-s001.zip › Figure S10.tif]

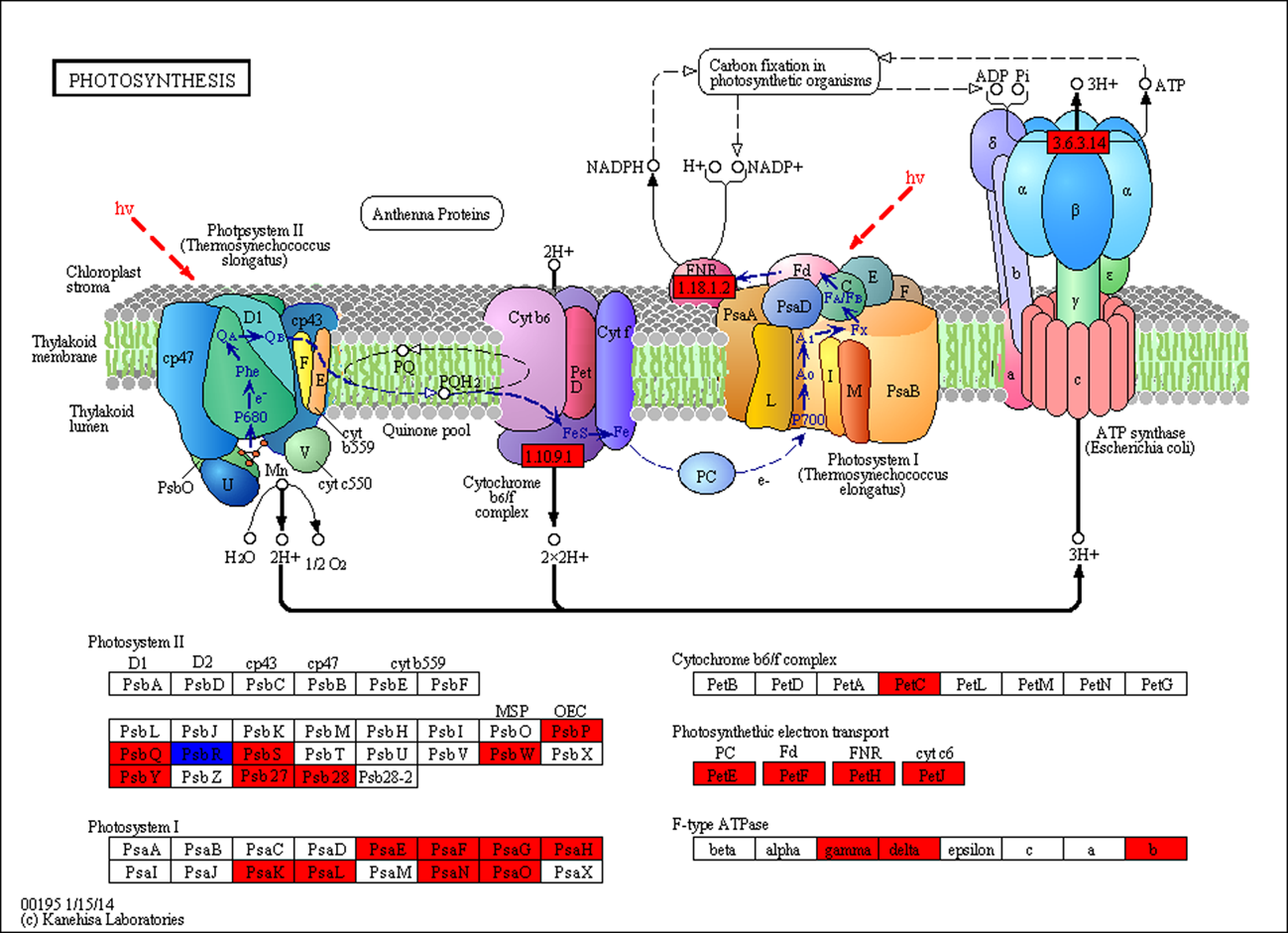

Supplement: Supplementary file 1 [file ijms-19-01324-s001.zip › Figure S11.tif]

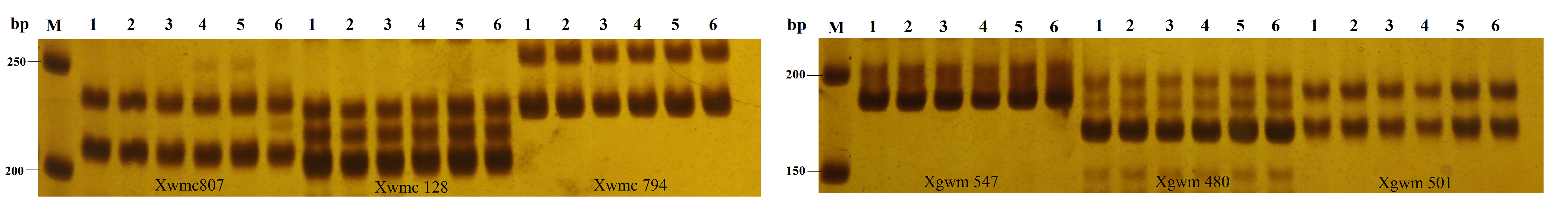

Supplement: Supplementary file 1 [file ijms-19-01324-s001.zip › Figure S2.tif]

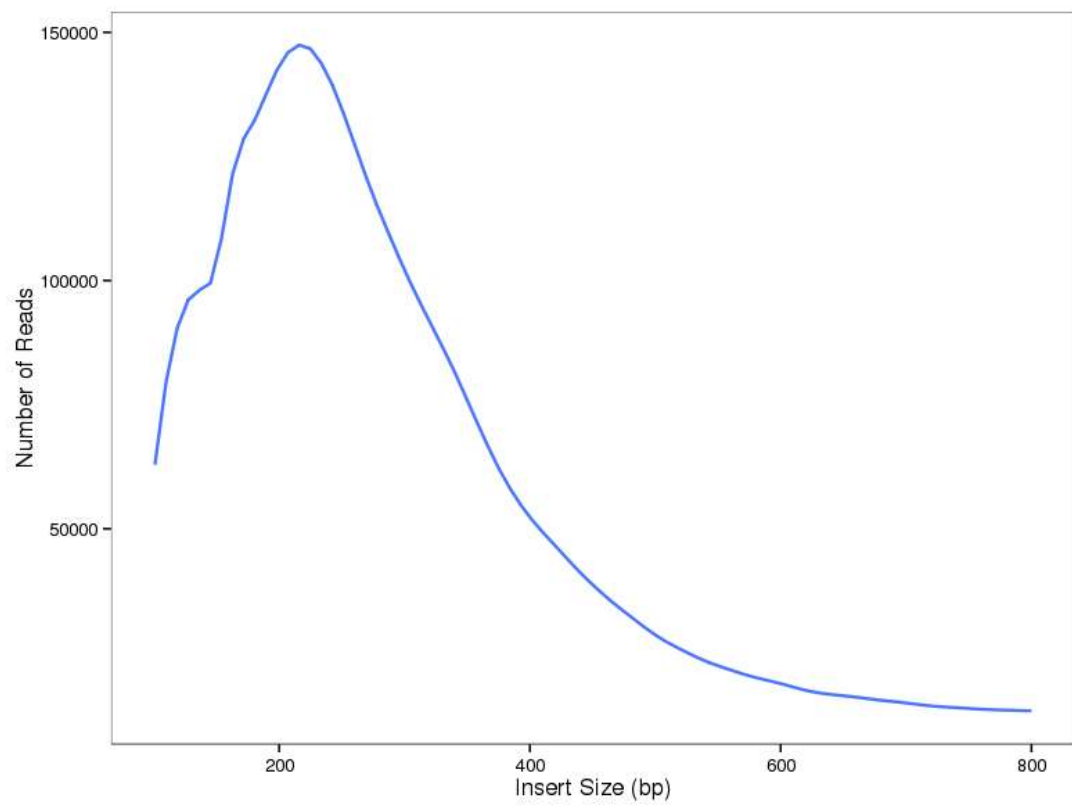

T1

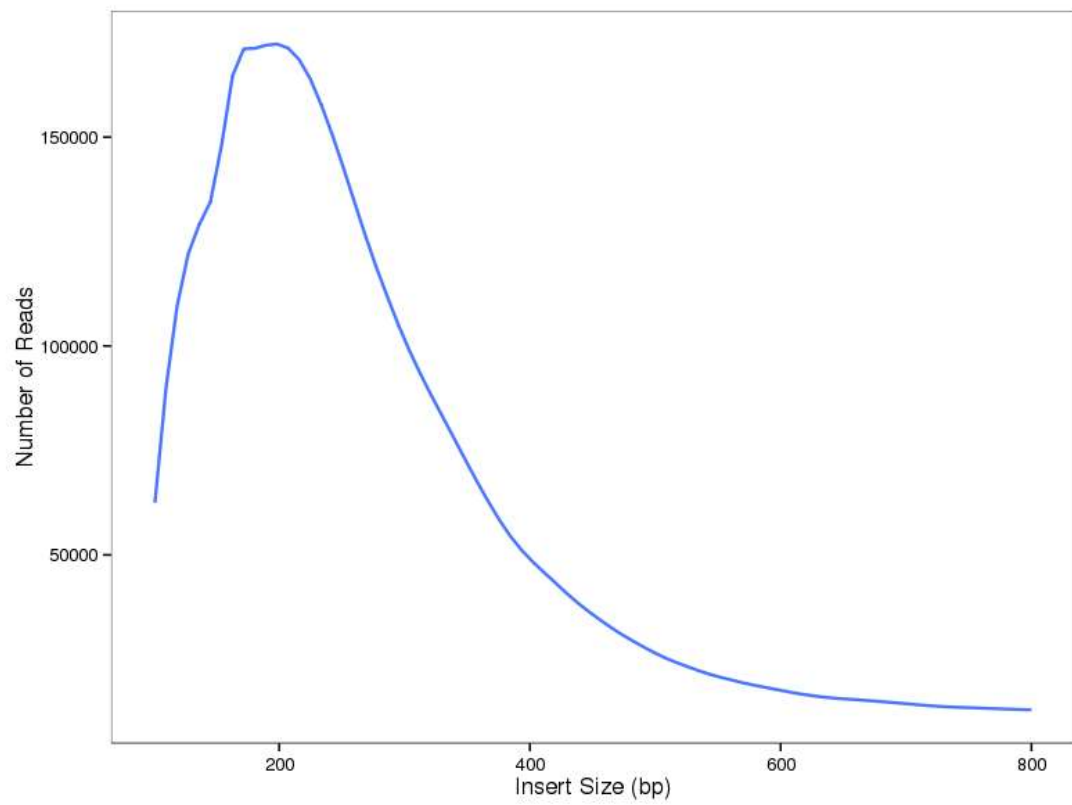

T2

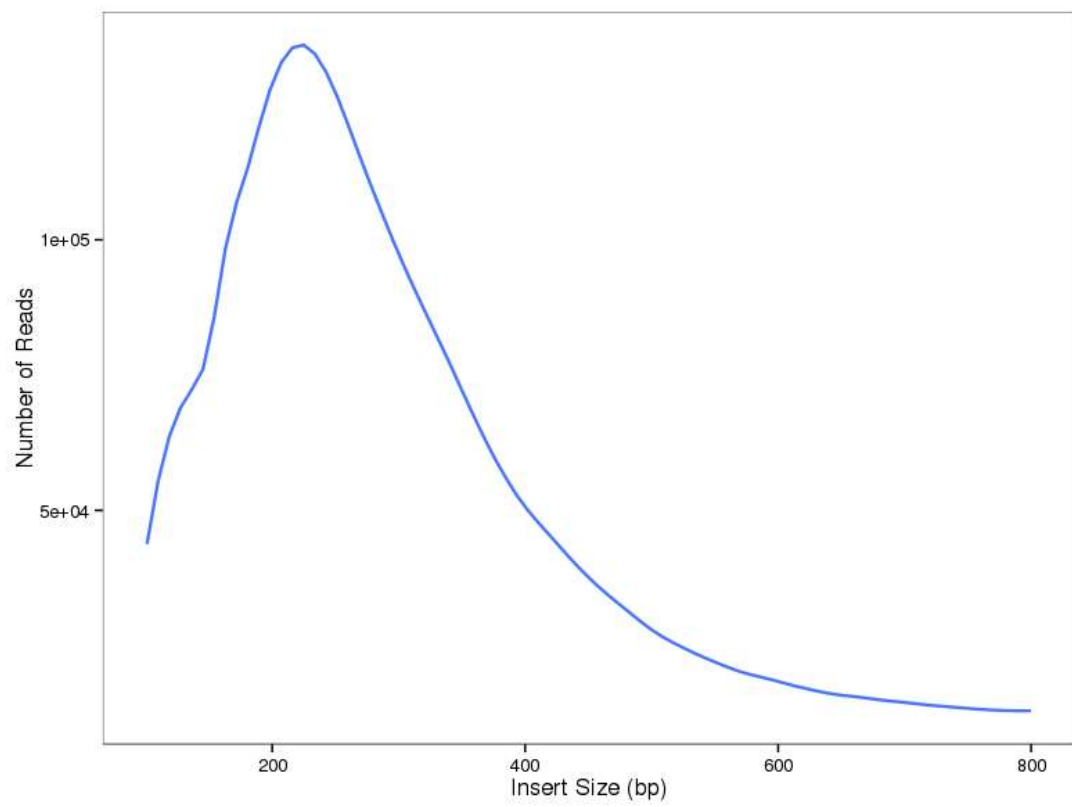

T3

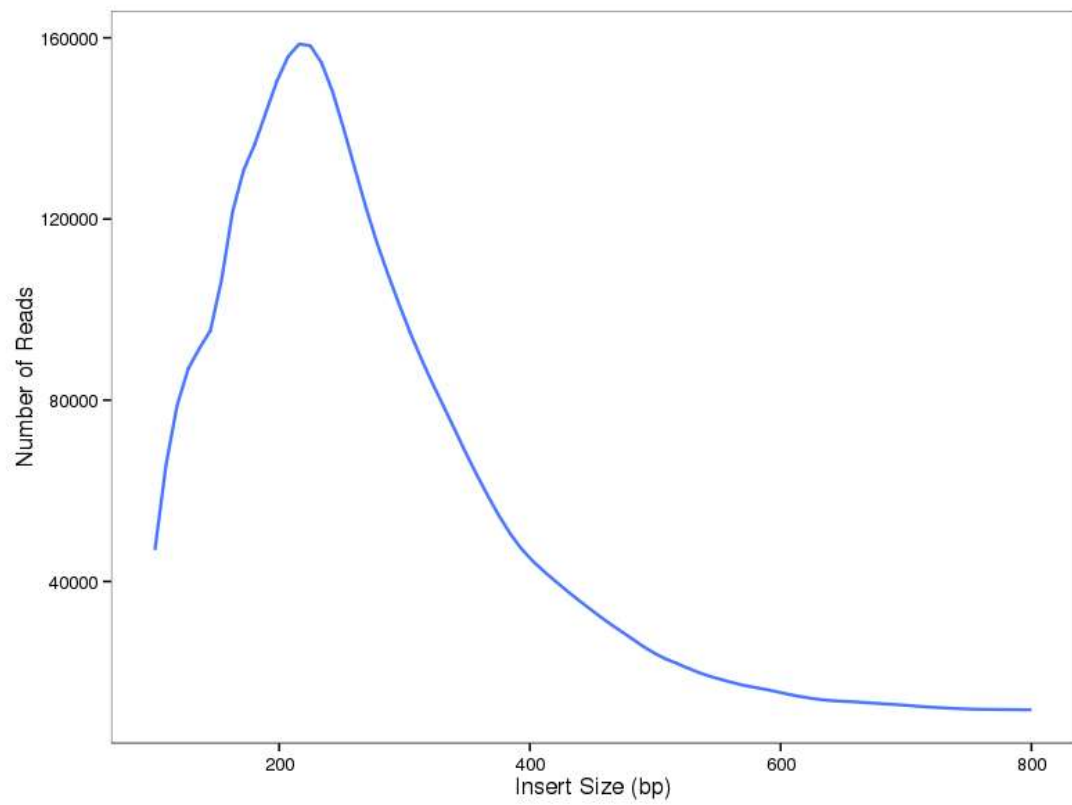

T4

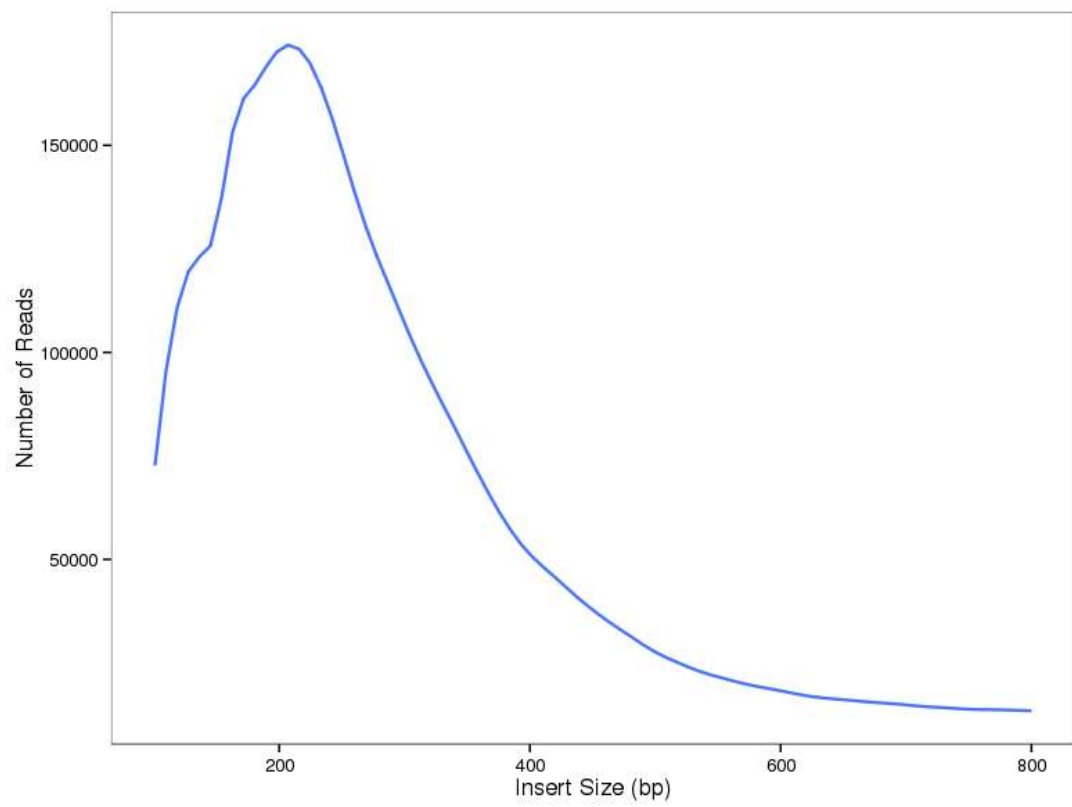

T5

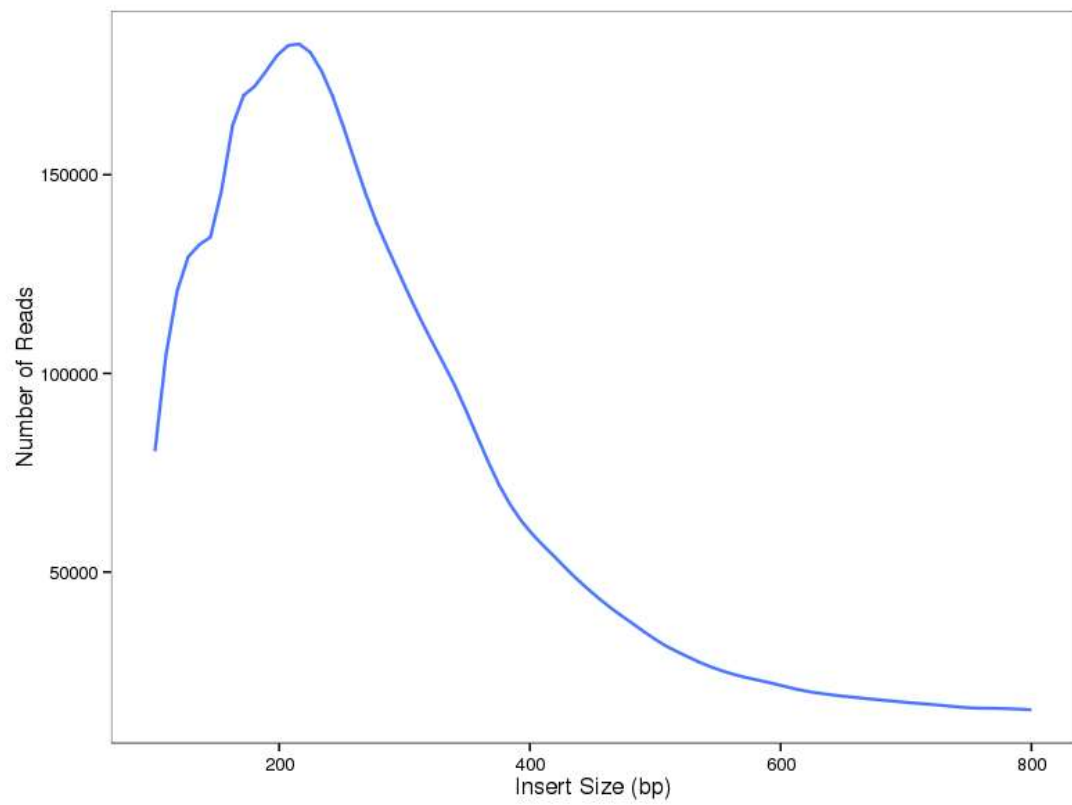

T6

Supplement: Supplementary file 1 [file ijms-19-01324-s001.zip › Figure S3.pdf]

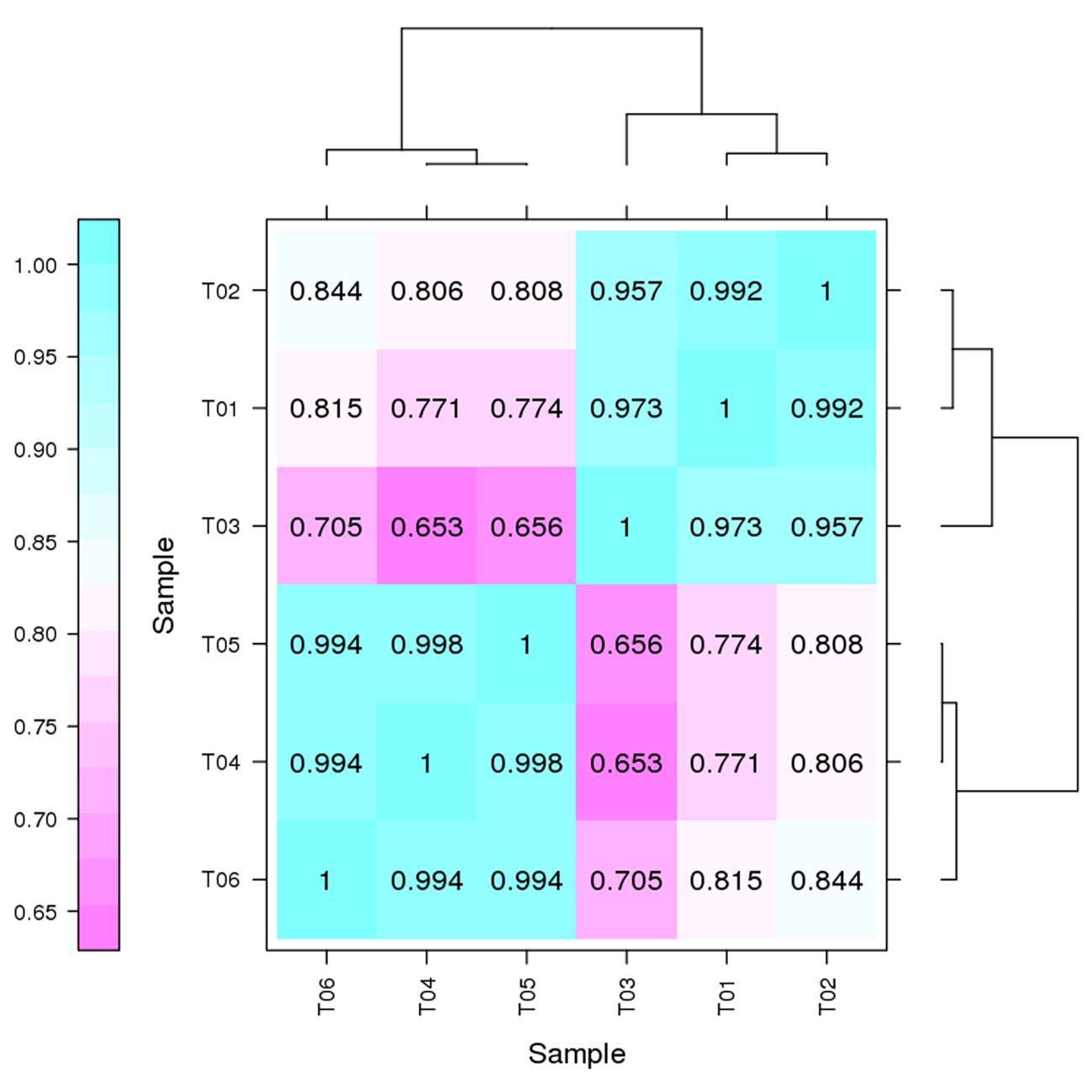

Supplement: Supplementary file 1 [file ijms-19-01324-s001.zip › Figure S4.tif]

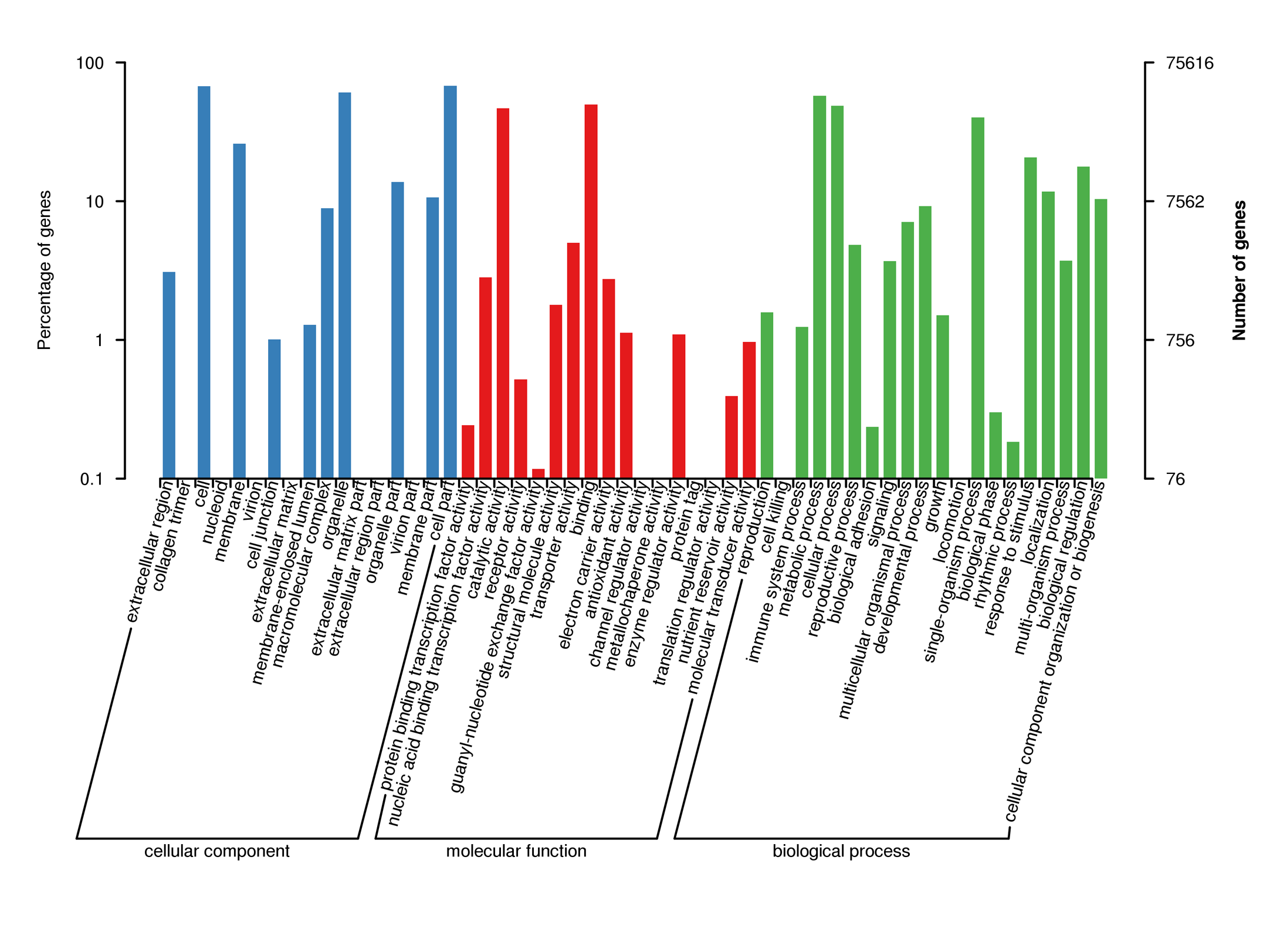

Supplement: Supplementary file 1 [file ijms-19-01324-s001.zip › Figure S5.tif]

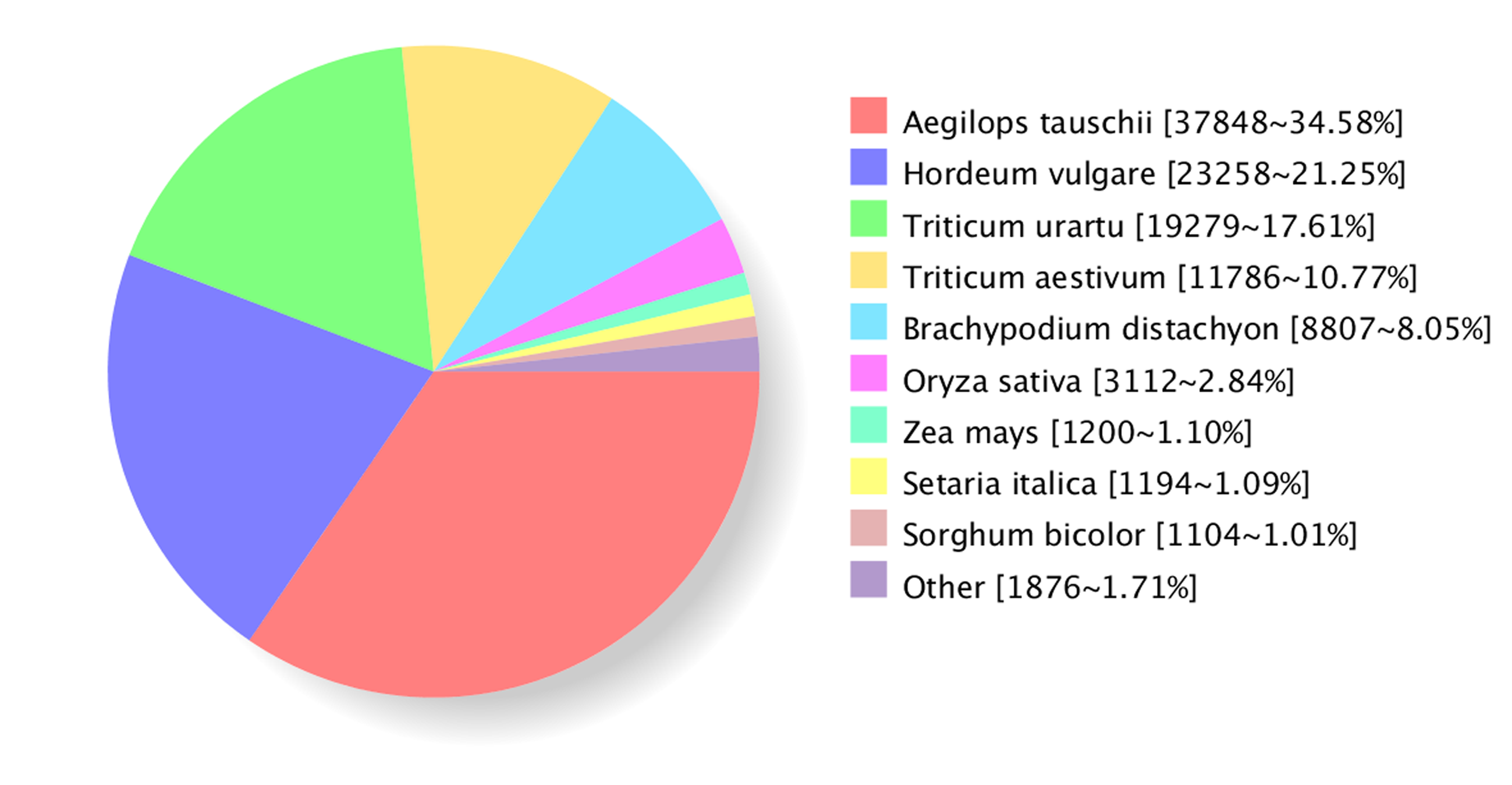

Supplement: Supplementary file 1 [file ijms-19-01324-s001.zip › Figure S6.tif]

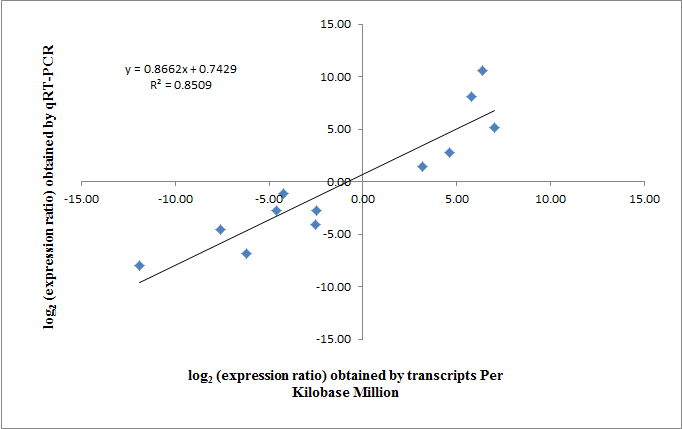

Supplement: Supplementary file 1 [file ijms-19-01324-s001.zip › Figure S7.tif]

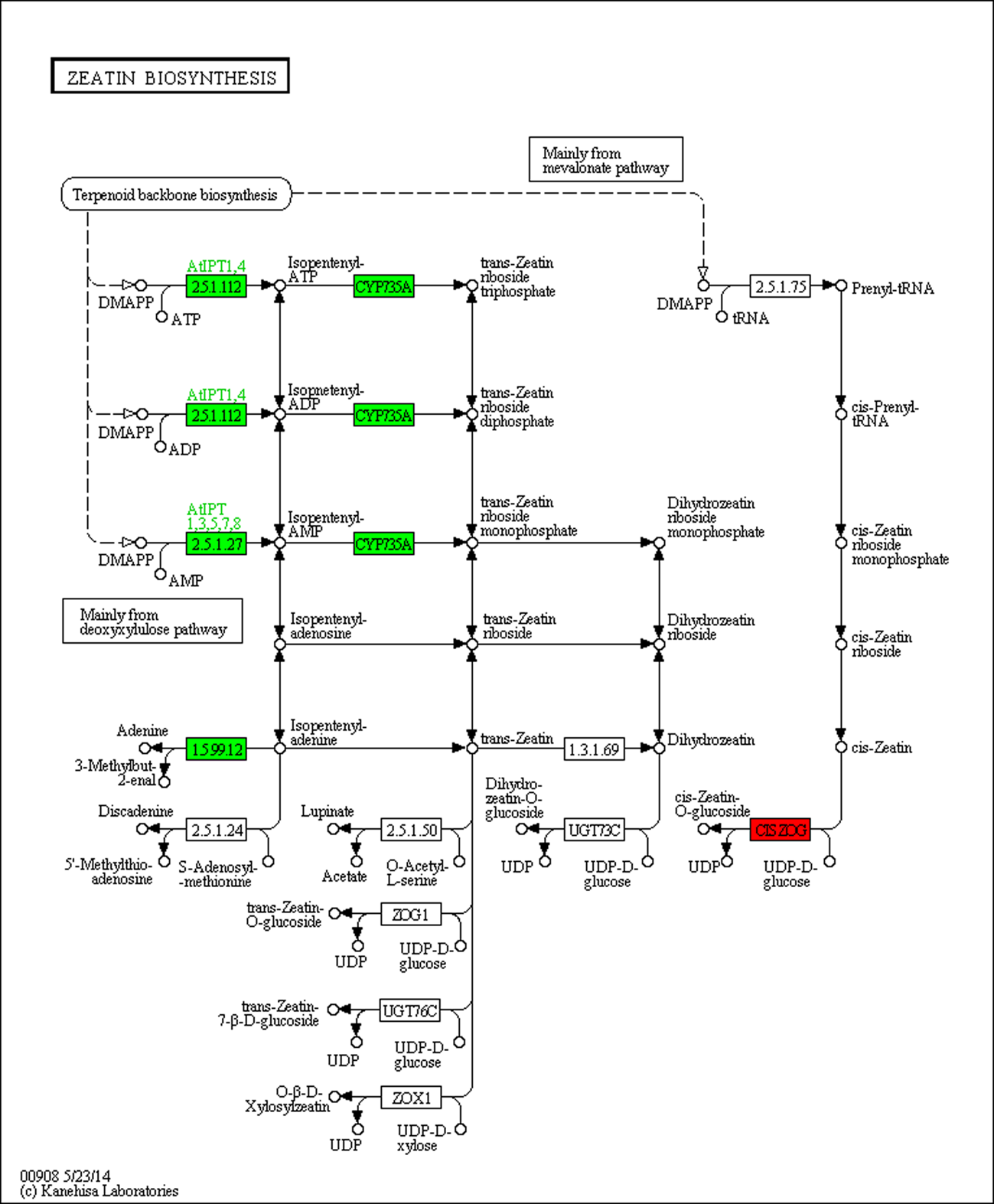

Supplement: Supplementary file 1 [file ijms-19-01324-s001.zip › Figure S8.tif]

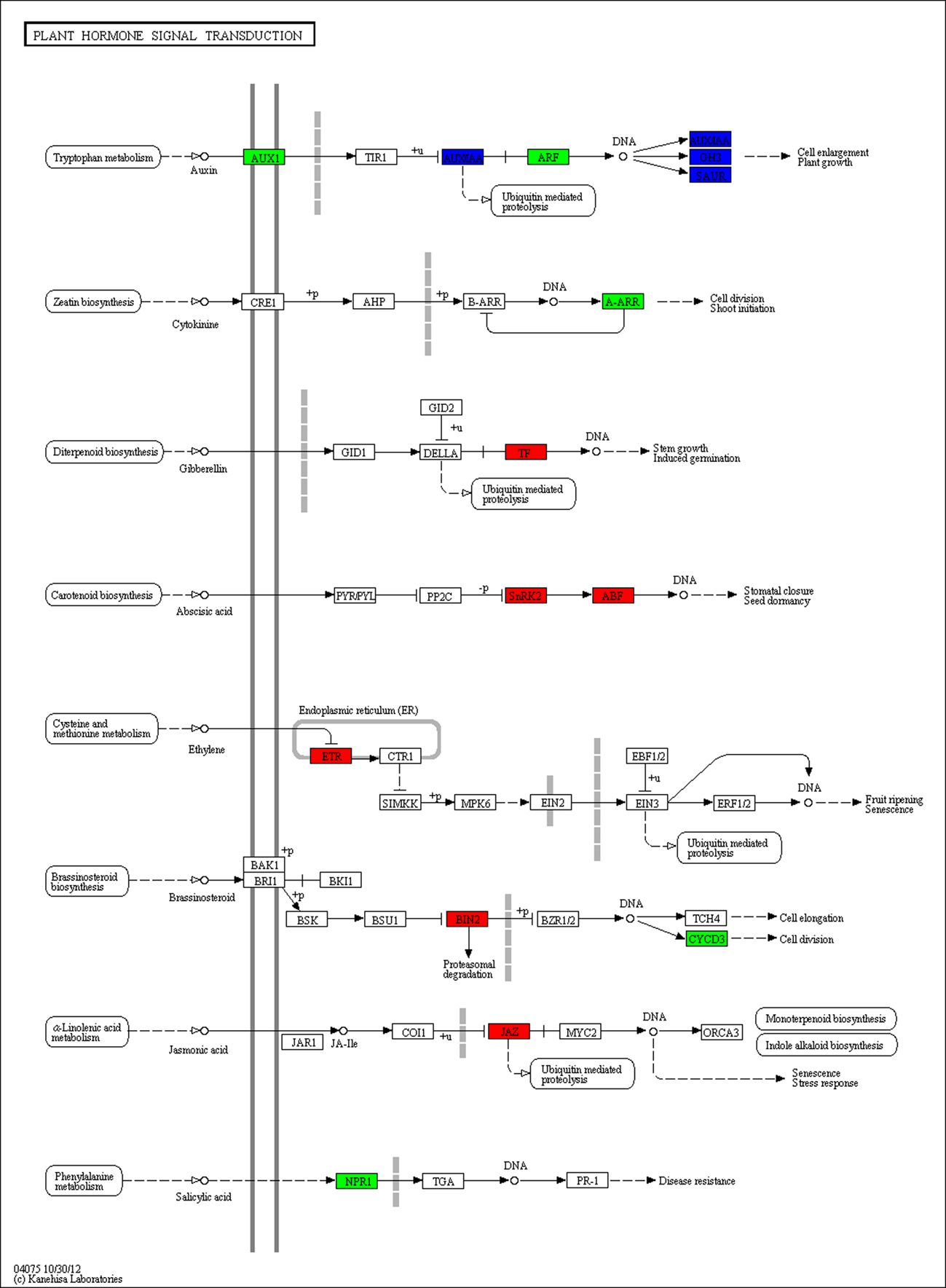

Supplement: Supplementary file 1 [file ijms-19-01324-s001.zip › Figure S9.tif]
